# Distinguishing between hot-spots and melting-pots of genetic diversity using haplotype connectivity

**DOI:** 10.1186/1748-7188-5-19

**Published:** 2010-03-20

**Authors:** Binh Nguyen, Andreas Spillner, Brent C Emerson, Vincent Moulton

**Affiliations:** 1 School of Computing Sciences, University of East Anglia, Norwich, NR4 7TJ, UK; 2 Department of Mathematics and Computer Science, University of Greifswald, 17489 Greifswald, Germany; 3 School of Biological Sciences, University of East Anglia, Norwich, NR4 7TJ, UK

## Abstract

We introduce a method to help identify how the genetic diversity of a species within a geographic region might have arisen. This problem appears, for example, in the context of identifying refugia in phylogeography, and in the conservation of biodiversity where it is a factor in nature reserve selection. Complementing current methods for measuring genetic diversity, we analyze pairwise distances between the haplotypes of a species found in a geographic region and derive a quantity, called haplotype connectivity, that aims to capture how divergent the haplotypes are relative to one another. We propose using haplotype connectivity to indicate whether, for geographic regions that harbor a highly diverse collection of haplotypes, diversity evolved inside a region over a long period of time (a "hot-spot") or is the result of a more recent mixture (a "melting-pot"). We describe how the haplotype connectivity for a collection of haplotypes can be computed efficiently and briefly discuss some related optimization problems that arise in this context. We illustrate the applicability of our method using two previously published data sets of a species of beetle from the genus *Brachyderes *and a species of tree from the genus *Pinus*.

## Background

It is now increasingly recognized that past climatic events have played a significant role in shaping the distribution of genetic diversity within species across the landscape. The distribution of this genetic diversity can leave signatures indicating the locations of refugia or "hot-spots", i.e. regions in which species have persisted for long periods of time. These regions are important as they have contributed to much of the observed structuring of genetic variation across the landscape [1].

**Figure 1:**
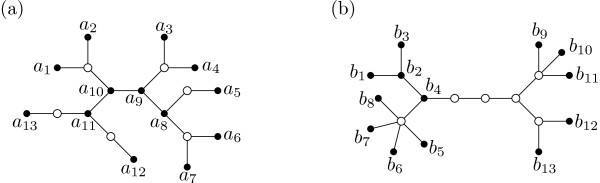
**Haplotype phylogenies**. Haplotype phylogenies for a collection *X *of haplotypes. (a) A hot-spot scenario, with *X *= {*a*_1_, ..., *a*_13_}. (b) A melting-pot scenario with *X *= {*b*_1_, ..., *b*_13_}. All edges have length 1.

It has been observed (e.g. [2,3]) that hot-spots may be distinguished by high levels of genetic diversity relative to the geographic domain that has been colonized from these regions. In particular, this provides a simple and intuitive diagnostic for identifying probable species refugia. However, the merging together of gene pools previously isolated in different refugia can also result in regions of high genetic diversity, so-called "melting-pots" [4]. Distinguishing between hot-spots and melting-pots is therefore an important problem in the area of phylogeography, where one of the main objectives is to identify the processes that are responsible for the contemporary geographic distributions of species. It is also a key issue in selecting nature reserves, where the aim is to choose regions in order to best conserve biodiversity (see e.g. [5]). Here we describe a new approach to help distinguish between hot-spots and melting-pots for a species that is based on the mutational properties of DNA sequences. Such sequences provide a robust framework for the assessment of historical relationships among genetic variants within a population. As a simple illustration of our approach, suppose that we sample a set *X *of DNA haplotypes from a species inhabiting a certain region. Consider the two hypothetical phylogenies for *X *in Figure 1, in which the vertices corresponding to the sampled haplotypes are given by black dots. Although the genetic diversity of *X *is the same according to the total length of both phylogenies, we see that in phylogeny (a) the haplotypes are dispersed across the phylogeny (a hot-spot scenario), whereas in phylogeny (b) the haplotypes form two groups (a melting-pot scenario) (cf. also category I vs category II patterns in [6]).

To differentiate between such behaviors, we introduce the concept of *haplotype connectivity *of a set *X *of haplotypes relative to a distance matrix *D *on *X*. This measure tries to quantify how well separated the haplotypes are relative to *D*. For distances *D *arising from path lengths in phylogenies where all edges have length 1 (i.e. the distance *D*(*x*, *y*) between two haplotypes *x*, *y *∈ *X *is simply the number of edges on the path from *x *to *y*) such as those presented in Figure 1, one can interpret the measure as follows. The haplotype connectivity of *X *relative to *D *is the smallest non-negative integer *c *so that, for any *x*, *y *∈ *X *that label vertices *u*, *v *in the tree, there is a sequence *u *= *w*_1_, *w*_2_, ..., *w*_*l *_= *v *of vertices in the tree such that (i) any two consecutive vertices in the sequence are adjacent and (ii) at least one of the vertices in every *c *consecutive vertices in the sequence is labeled by some element in X. For example, it can be checked that the phylogeny in (a) has haplotype connectivity 2, whereas the haplotype connectivity of the phylogeny in (b) is 5. In particular, the lower haplotype connectivity score corresponds to the hot-spot scenario.

To efficiently compute the haplotype connectivity of a collection of haplotypes *X *relative to a distance matrix *D*, we show how to make use of an algorithm for a related problem presented in [7]. In addition, for fixed *k*, we develop some algorithms for finding the minimum/maximum haplotype connectivity of any subset of *X *of size *k*. As we shall see, this allows us to more easily compare the haplotype connectivity of different size sets as it takes the sample-size bias into account.

Our new method complements the approach presented in [8] for detecting zones of secondary contact (i.e. melting-pots) based on nested clade analysis [9,10] (it should, however, be noted that there is some debate in the literature concerning the validity of nested clade analysis [11]). It is also related to the method for inferring population genetic processes based on the frequency distribution of pairwise distances between haplotypes presented in [12] (see also [13,14]). To the best of our knowledge, these are currently the main computational approaches used to distinguish hot-spots and melting-pots based on molecular sequence data.

The rest of the paper is organized as follows. In the next section we formally define haplotype connectivity, show how this quantity can be computed efficiently and discuss some optimization problems that naturally arise in the context of this paper. We then illustrate the applicability of our method using two published data sets encompassing different spatial scales, before concluding with a short discussion of possible future directions.

## Methods

We now describe our new methods. We assume that we are given a set *X *of haplotypes, together with a dissimilarity measure *D *on *X *that quantifies the genetic distance *D*(*x*, *y*) between every pair *x*, *y *in *X*. There are several dissimilarity measures for DNA haplotypes, such as the Hamming distance or the phyletic distance, that is, the distance relative to a phylogeny on *X *(see e.g. [12,15-17]).

### Haplotype connectivity

Given a subset *Y *of *X *(corresponding, for example, to the haplotypes that are found in some given region), we aim to quantify how difficult it is relative to *D *to link any pair *x*, *y *∈ *Y *by a sequence of intermediate haplotypes also belonging to *Y*.

To do this, we shall use the concept of a threshold graph (see e.g. [18,19]). For a non-negative number or *threshold t*, we define the graph *G*_*t*_(*Y*) with vertex set *Y *and edge set consisting of those pairs of distinct haplotypes *x*, *y *∈ *Y *with *D*(*x*, *y*) ≤ *t*. In addition we assign to every edge *e *= {*x*, *y*} the weight ω (*e*) := *D*(*x*, *y*). The *haplotype connectivity *of *Y *(relative to *D*) is then defined to be the smallest number *t *such that the graph *G*_*t*_(*Y*) is connected (as usual, the graph *G*_*t*_(*Y*) is connected if there is some path in *G*_*t*_(*Y*) between any pair of elements in *Y*). We denote this number by *HC*(*Y*, *D*) or just *HC*(*Y*) in case it is clear what *D *is from the context.

**Figure 2:**
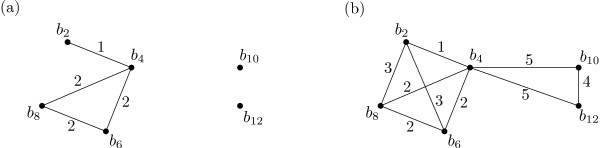
**Threshold graphs**. The graphs (a) *G*_2_(*X*) and (b) *G*_5_(*X*) induced by the haplotype phylogeny in Figure 1(b) on the subset *Y *= {*b*_2_, *b*_4_, ..., *b*_12_}. For example, there is no edge joining the haplotypes *b*_2 _and *b*_10 _in *G*_2_(*X*) because the number of edges on the path from *b*_2 _to *b*_10 _in the phylogeny is greater than 2.

To illustrate these definitions, consider the subset *Y *= {*b*_2_, *b*_4_, ..., *b*_12_} of *X *= {*b*_1_, *b*_2_, ..., *b*_13_} and the phyletic distance *D *on *X *induced by the phylogeny in Figure 1(b), i.e. the distance obtained by taking the path length between pairs of haplotypes. In Figure 2(a) we depict the graph *G*_2_(*Y*). This is not connected, and so *HC*(*Y*) > 2. However, it is straight-forward to check that *HC*(*Y*) = 5 (the graph *G*_5_(*Y*) is depicted in Figure 2(b)).

Now, if *t *≥ max{*D*(*x*, *y*): *x*, *y *∈ *Y *} then the graph *G*_*t*_(*Y*) is the complete graph on *Y*, i.e. the graph in which every pair of vertices is linked by an edge, which we denote by G_*_(*Y*). For every *spanning tree T *of G_*_(*Y*), that is, a subgraph of G_*_(*Y*) that is a tree and that contains all vertices of G_*_(*Y*), let ω_*max*_(*T*) be the maximum of the edge weights over all edges of *T*. We claim that

Indeed, since every spanning tree *T *of G_*_(*Y*) is a connected subgraph of G_*_(*Y*), we must have *HC*(*Y*) ≤ *ω*_*max*_(*T*). Conversely, putting *t *:= *HC*(*Y*), by definition of *HC*(*Y*) the graph *G*_*t*_(*Y*) is connected and every spanning tree *T *of *G*_*t*_(*Y*) is also a spanning tree of G_*_(*Y*) with *ω*_*max*_(*T*) ≤ *t *= *HC*(*Y*). In particular, this implies that there always exist *x*, *y *∈ *Y *with *HC*(*Y*) = *D*(*x*, *y*). A spanning tree *T *of G_*_(*Y*) with score ω_*max*_(*T*) equal to *HC*(*Y*) is also known as a *bottleneck minimum spanning tree *or *bottleneck MST*, for short. In [7] an algorithm for computing such a tree is presented. This algorithm performs a *binary search *[[20], p. 37] on the edge weights in the graph. However, rather than explicitly sorting these weights first, an algorithm for finding the median [21] is used. In this way, since in each step of the binary search at least half of the remaining edges in the graph can be discarded, the overall run time is *O*(*m*) for a connected edge-weighted graph with *m *edges. Thus, as G_*_(*Y*) has *O*(|*Y*|^2^) edges, *HC*(*Y*) can be computed in *O*(|*Y*|^2^) time, which is clearly optimal. Alternatively, since every *minimum spanning tree (MST)*, that is, a spanning tree of G_*_(*Y*) with minimum total edge weight, can easily be seen to be a bottleneck minimum spanning tree of G_*_(*Y*), one can also employ any algorithm for finding an MST of G_*_(*Y*) (see e.g. [[20], ch. 23]) to compute *HC*(*Y*).

### Maximizing and minimizing haplotype connectivity

In our analyses it can be helpful to understand how large *HC*(*Y*) is relative to other subsets of the same size as *Y*. Therefore, for a subset *Y *⊆ *X *of *k *haplotypes, we now consider the problem of computing the minimum and maximum possible haplotype connectivity, denoted by *HC*_min_(*k*) and *HC*_max_(*k*), respectively, over all subsets of *X *containing precisely *k *haplotypes. Based on this, we define, for any subset *Y *⊆ *X*, the *normalized haplotype connectivity of Y *by

Note that this normalized score always lies between 0 and 1. We will use this score to rank regions according to their haplotype connectivity.

Computing *HC*_min_(*k*) amounts to finding a *k*-element subset *Z *of *X *such that the score ω_*max*_(*T*) of a bottleneck MST *T *of G_*_(*Z*) is minimized. This problem is known as the *bottleneck k*-*MST problem *and can be solved in optimal *O*(|*X*|^2^) time by extending the algorithm for computing a bottleneck MST mentioned in the previous section [22]. The key idea is to introduce, in addition to the given weights on the edges, a suitable weighting of the vertices of the graph.

To compute *HC*_max_(*k*), we first note that this quantity equals the smallest threshold *t *such that for every subset *Z *of *X *with *k *elements the graph *G*_*t*_(*Z*) is connected. In other words, every *vertex separator *of *G*_*t*_(*X*) (i.e. every subset *S *of *X *with *G*_*t*_(*X *- *S*) disconnected) must have more than |*X*| - *k *elements.

Several algorithms for computing a vertex separator of minimum size are known, e.g. based on a reformulation as a *network flow *problem [23]. The currently fastest algorithm for this problem employs so-called *expander graphs *and runs in *O*(*sn*^2 ^+ ) time for a graph with *n *vertices and *m *edges, where *s *is the minimum size of a vertex separator [24]. Hence, by performing a binary search over the increasingly sorted list of values *D*(*x*, *y*), *x*, *y *∈ *X*, *HC*_max_(*k*) can be computed in *O*( log *n*) time.

### Measuring and optimizing genetic diversity

As mentioned in the introduction, we are particularly interested in regions with a high level of genetic diversity. Therefore, as part of our analyses, it is necessary to measure the genetic diversity of any subset *Y *⊆ *X *of DNA haplotypes. There are several measures commonly used for this - for example, the number and frequency of haplotypes (see e.g. [15]) or the number of segregating sites found in the haplotypes (see e.g. [25]). However, in our studies we found that it made little difference to our results which method was used (data not shown).

As with the haplotype connectivity measure, for the purposes of comparing the diversity of samples having different sizes, it can be useful to compute how large the genetic diversity of a subset *Y *is relative to other subsets of the same size. Whether or not this can be done efficiently obviously depends on how the measure of genetic diversity is defined. For the purposes of illustration we now describe how this may be done for two common measures of genetic diversity, which we shall also use in our examples below.

In case a phylogeny is available, we can make all of our computations (including those for haplotype connectivity) relative to the genetic distance *D *given by taking the phyletic distance. In this situation, the genetic diversity of *Y *relative to *D *is commonly defined as the total length of the restriction of the phylogeny to *Y *(i.e. the length of the shortest subtree spanned by the elements in *Y*) which we denote by *PD*(*Y*). This measure has been used in the analysis of intraspecific patterns (see e.g. [26,27]) and also in interspecific studies (see e.g. [28]), in which it is commonly known as the *phylogenetic diversity (PD) *measure.

We denote the minimum and maximum of *PD*(*Y*) over all subsets *Y *⊆ *X *of size *k *= |*Y*| by *PD*_min_(*k*) and *PD*_max_(*k*), respectively. Interestingly, both of these quantities can be computed efficiently: For *PD*_max_(*k*) there is a simple greedy algorithm [29,30] that can be implemented to run in *O*(|*X*|) time [31]. For *PD*_min_(*k*) a polynomial time algorithm based on dynamic programming is described in [32] in the context of the so-called *i*-*tree problem*. Implementations of efficient algorithms for computing these quantities are available online [33]. Therefore, one can also compute in polynomial time the normalized score PD*(*Y*), which is defined, for any subset *Y *⊆ *X*, analogously to HC*(*Y*) above, by

In case solely a distance matrix *D *is available, a common measure of the genetic diversity of a set *Y *relative to *D *is (up to a constant scaling-factor) the average squared pairwise distance between elements in *Y *[34], i.e. , where  denotes the set of all 2-element subsets of *Y*. The normalized score AD*(*Y*) is defined in a completely analogous way to the scores HC*(*Y*) and PD*(*Y*) above. However, in contrast to *PD*(*Y*), given *D *and *k*, it is NP-hard to compute either the minimum or maximum diversity score, denoted by *AD*_min_(*k*) and *AD*_max_(*k*), respectively, over all subsets of *X *with *k *elements. Indeed, the maximization problem, which is also known as the *MAXISUM facility dispersion problem*, is shown to be NP-hard in [35], and the minimization problem can be shown to be NP-hard using similar arguments. However, we note that there are algorithms that can solve instances of the maximization problem for |*X*| ≤ 60 usually within seconds on a modern desktop PC (see e.g. [36]).

## Results and discussion

To illustrate the applicability of our approach we apply it to two previously published data sets that were analyzed in [37] and [17], respectively.

### Beetle Data

**Figure 3:**
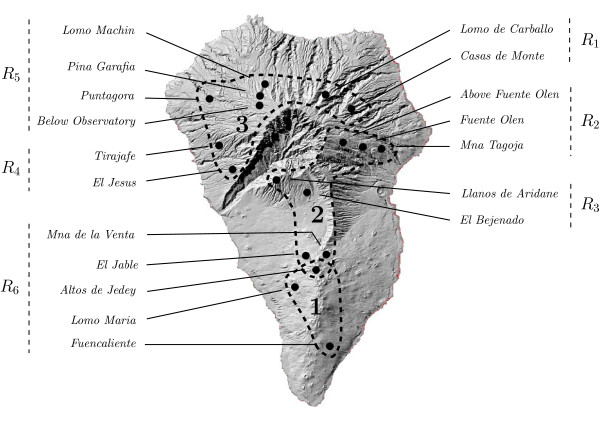
**Sampling locations and regions for beetle data**. A map of La Palma with sampling locations indicated by black dots [37]. Sampling locations where haplotypes from a particular phylogroup (cf. Figure 4) were found are depicted by the dashed curves. Note that the sampling location *Altos de Jedey *is the only one where haplotypes from two distinct phylogroups (namely 1 and 2) were found. The six groups of sampling locations corresponding to the six regions *R*_1_, *R*_2_, ..., *R*_6 _discussed in the text are also indicated.

**Figure 4:**
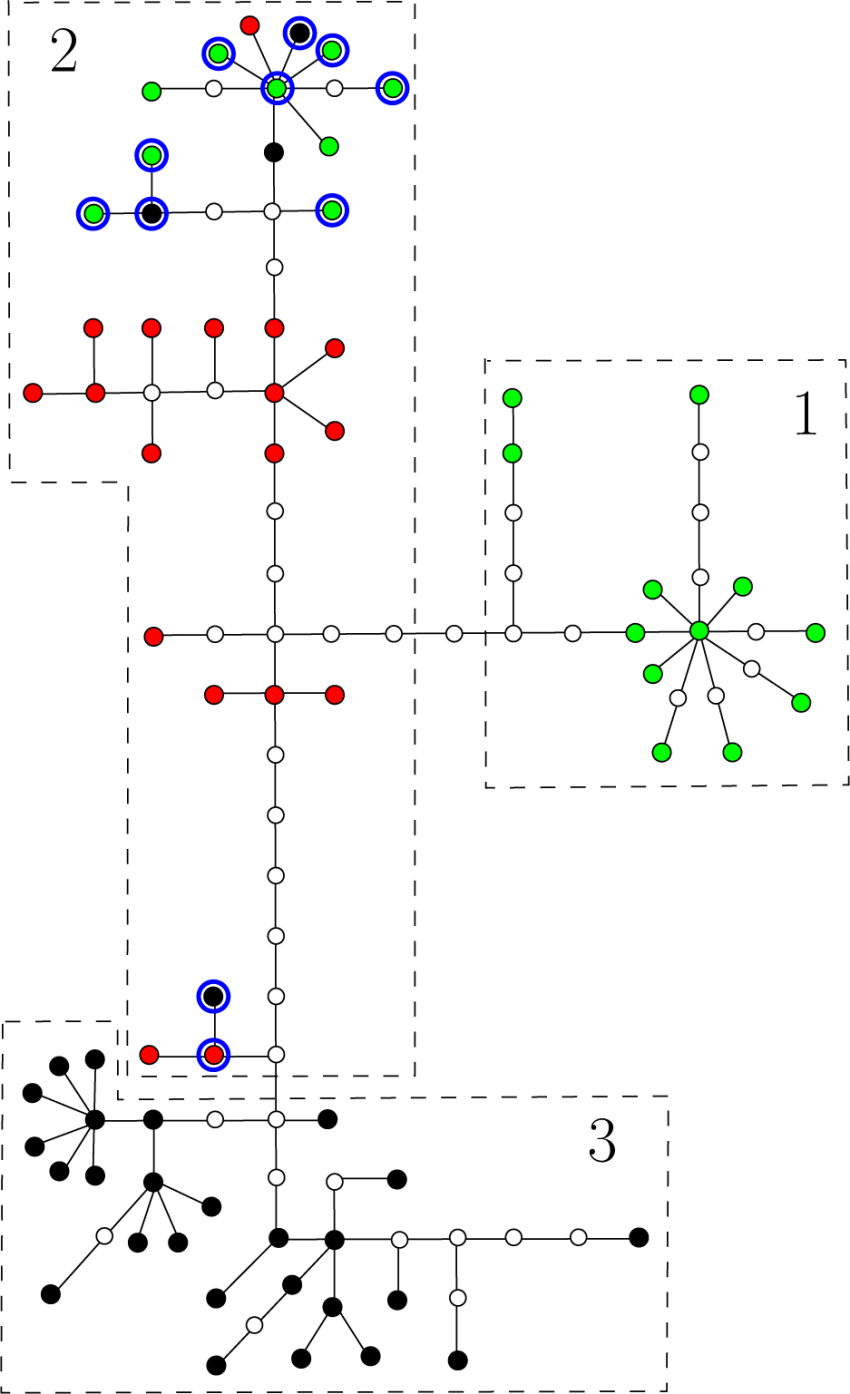
**Haplotype phylogeny for beetle data**. The haplotype network presented in [37] for the haplotypes collected in La Palma. Note that all edges have length 1. The colored dots (black, red and green) represent the sampled haplotypes and the white dots hypothetical intermediates. Dashed boxes correspond to the three phylogroups, 1-3, identified in [37]. The haplotypes found in region *R*_2 _are highlighted in red, those found in *R*_6 _in green and those found in *R*_3 _are indicated by blue circles.

The first data set was used as part of a phylogeographic study of the beetle species *Brachyderes rugatus rugatus *on La Palma (Canary Islands) [37]. In this study 138 individual beetles were sampled. The 18 sampling locations are shown in Figure 3. Using sequence data from the mitochondrial COII gene (for details see [37]), the 138 samples were subsequently grouped into 69 haplotypes, and a haplotype phylogeny based on the parsimony criterion was constructed using the TCS program [38]. This phylogeny is presented in Figure 4.

According to this phylogeny, the haplotypes were divided into 3 phylogroups, as indicated on the phylogeny and in Figure 3. Based on these groupings it was concluded for *Brachyderes rugatus rugatus *that (i) there is a region of secondary contact, or melting-pot, in the South of the island at the overlap of regions 1 and 2, and (ii) that there is an ancestral region or hot-spot in the region containing the three sampling locations in the top right of region 2. Note that in [37] support for conclusion (i) was provided by performing the test given in [8] for detecting zones of secondary contact, which essentially involves calculation of the average distance between the geographic centers of clades at increasing nesting levels in a phylogeny on the haplotypes of interest.

To investigate whether our new method was supportive of conclusions (i) and (ii) or not, we first grouped the sampling locations into 6 regions *R*_1_, ..., *R*_6 _as shown in Figure 3. We used these regions rather than the individual sampling locations, since the number of samples taken at each location was very small (between 2 and 8). When forming the groups, geographically close locations were grouped together. We also considered other groupings based on geographic proximity (data not shown) and the outcome was similar, though less pronounced when the number of groupings was reduced (smallest number of groupings used was 3). We then measured the diversity (using the measure *PD*) and haplotype connectivity for the haplotypes found in each region *R*_*i *_relative to the phyletic distances given by the phylogeny in Figure 4, as described in the Methods section.

The results for the 6 regions are summarized in Table 1. In this table, we present the size of the subset *Y *of haplotypes found in the region (column 2), the values *PD*(*Y*), *PD*_min_(|*Y*|), *PD*_max_(|*Y*|) (columns 3-5), and the normalized diversity score PD*(*Y*) (column 6) as defined in the Methods section. Similarly, we present the values *HC*(*Y*), *HC*_min_(|*Y*|), *HC*_max_(|*Y*|) and HC*(*Y*) (columns 7-10).

**Table 1:** Scores for beetle data.

Region	Number of Haplotypes in region	Diversity				Haplotype connectivity			
		*PD*	*PD*_min_	*PD*_max_	PD*	*HC*	*HC*_min_	*HC*_max_	HC*
*R*_6_	21	47	25	87	0.35	14	3	25	0.50
*R*_3_	11	28	10	67	0.32	16	1	27	0.58
*R*_2_	18	33	20	81	0.21	7	3	25	0.18
*R*_4_	7	14	6	55	0.16	5	1	27	0.15
*R*_5_	18	29	20	81	0.15	5	3	25	0.09
*R*_1_	5	10	4	48	0.14	7	1	28	0.22

Diversity and haplotype connectivity scores for the geographic regions on La Palma indicated in Figure 3, ranked according to normalized phylogenetic diversity scores, PD*, as defined in the main text. The columns labeled with *PD*_min_, *PD*_max_, *HC*_min _and *HC*_max _contain the minimum/maximum score over all subsets containing the same number of haplotypes as found in the region.

As can be seen in Table 1, the two regions with the highest PD*score are *R*_6 _and *R*_3_, which also have a much higher HC* score than any of the other four regions. This is supportive of conclusion (i), i.e. that *R*_6 _is probably a melting-pot. Indeed, in Figure 4 the haplotypes found in region *R*_6 _are highlighted in green, and it can be seen that they clump together into two groups. This also indicates why we obtained a high HC* score for this region. Similarly, the high PD* and HC* scores for region *R*_3 _suggests that this region is a melting-pot as well, a conclusion that is consistent with the findings in [37] where it is suggested that in *R*_3 _range expansions toward the South and the Northwest partially overlapped.

Concerning conclusion (ii), we see that amongst the remaining regions *R*_2 _clearly has the highest PD* score and a much lower HC* score than *R*_6 _and *R*_3_. This pattern of scores, i.e. relatively high diversity and low haplotype connectivity, is more supportive of a hot-spot scenario rather than a melting-pot scenario, in agreement with conclusion (ii). Examining Figure 4, we see that the haplotypes in *R*_2 _(highlighted in red) are relatively spread out over the haplotype phylogeny, hence the low haplotype connectivity score.

### Pine Data

**Figure 5:**
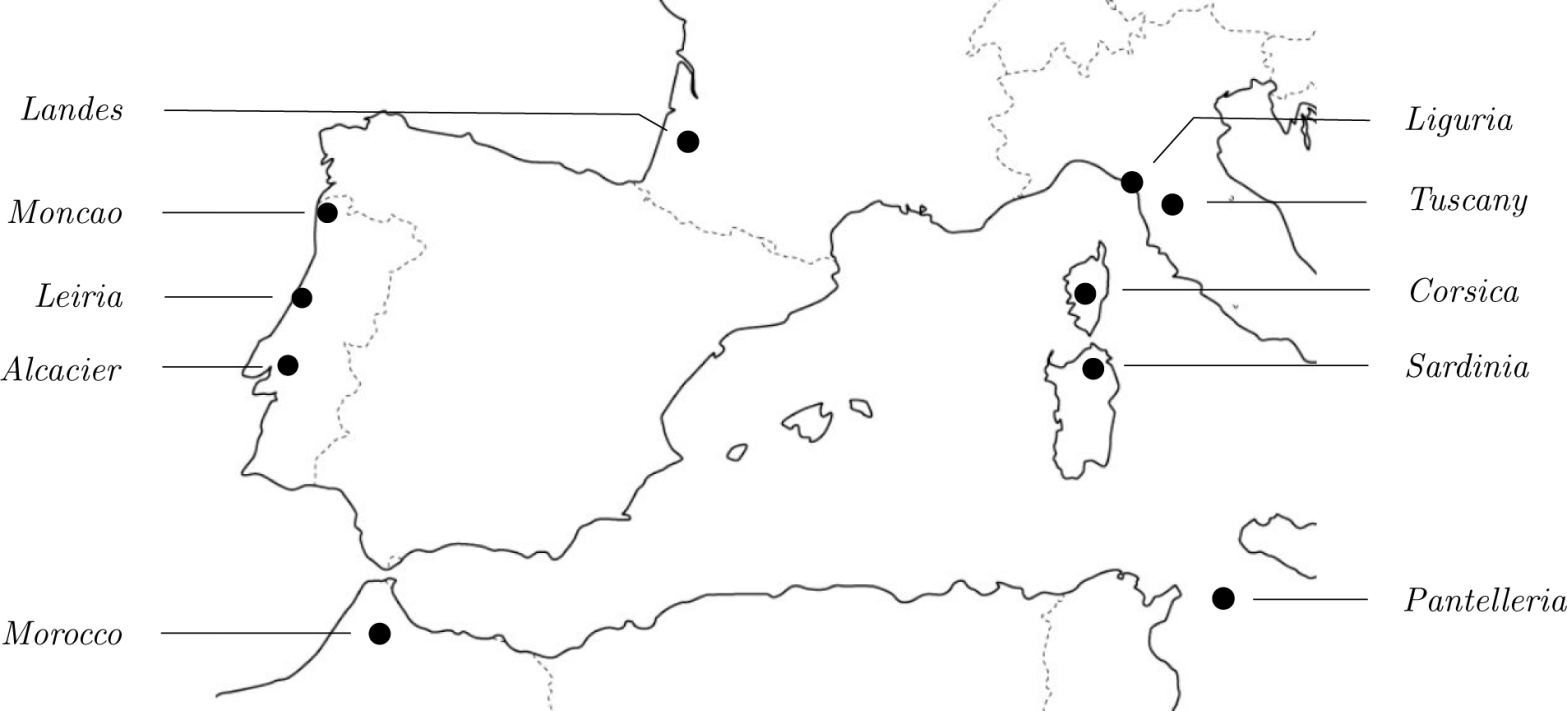
**Sampling locations for pine data**. Sampling locations for the data set in [17].

The second data set that we consider formed part of a study of the phylogeographic history of the species *Pinus pinaster *around the Mediterranean [17]. Samples were taken from 10 locations as indicated in Figure 5. Sequence data consisting of nine chloroplast simple sequence repeat markers gave rise to 34 different haplotypes (for details see [17]). For these 34 haplotypes a distance matrix was computed using the pairwise haplotypic difference (that is, for any two haplotypes, the sum of the difference between the allele size over the nine loci).

**Figure 6:**
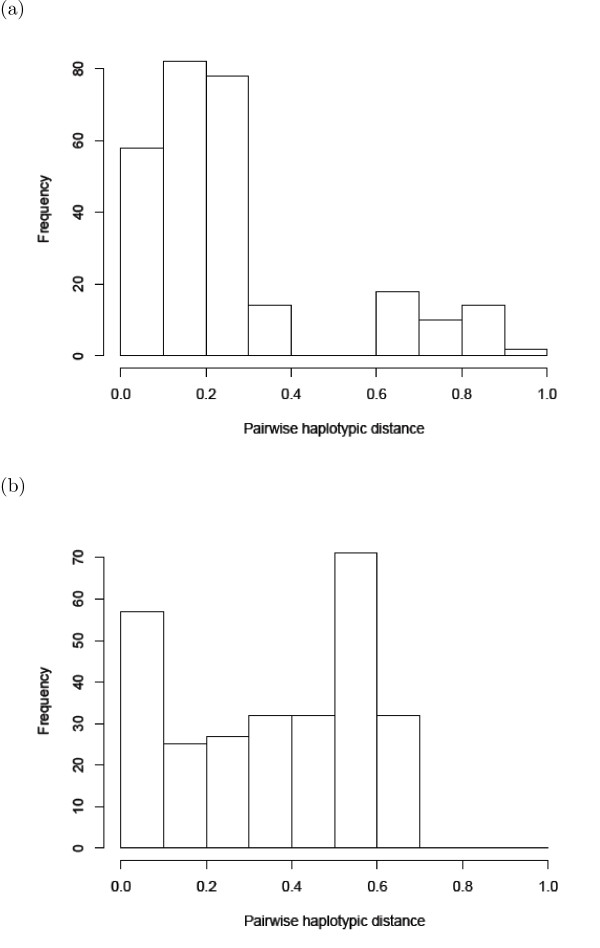
**Genetic diversity spectrum**. The genetic diversity spectrum (GDS) for (a) the Landes location and (b) the Pantelleria location in Figure 5. For every possible distance, the number of pairs of haplotypes that are that distance apart is depicted.

To understand the phylogeographic structure of this data, in [17] the frequency distribution of the pairwise distances between haplotypes, sometimes also called the *genetic diversity spectrum *(GDS) [12], was computed. We have recomputed this and depict the result in Figure 6. In particular, based on considerations - such as the shape of the GDS for the Landes and Pantelleria locations - it was hypothesized that Landes and Pantelleria are hot-spots, although it was also stated that the hypothesis that they are melting-pots could not be excluded [[17], p.462]. Indeed, in a more recent extended phylogeographic study of *Pinus pinaster *[39] it was concluded that Landes was more likely to be a melting-pot.

Using the same distance matrix, we computed diversity and haplotype connectivity scores for each of the 10 sampling locations as explained in the Methods section (using the measure *AD *for diversity). These are presented in Table 2. Note that, in contrast to [17], our scores do not take into account how often a haplotype was found in a particular location but rather which haplotypes were found.

**Table 2:** Scores for pine data.

Sampling location	Number of Haplotypes in region	Diversity				Haplotype connectivity			
		*AD*	*AD*_min_	*AD*_max_	AD*	*HC*	*HC*_min_	*HC*_max_	HC*
Landes	6	2.45	0.33	7.14	0.31	6	1	10	0.56
Pantelleria	9	1.67	0.37	5.66	0.25	3	1	10	0.22
Leiria	8	0.73	0.36	6.06	0.06	1	1	10	0.00
Sardinia	9	0.70	0.37	5.66	0.06	2	1	10	0.11
Morocco	8	0.69	0.36	6.06	0.06	1	1	10	0.00
Corsica	8	0.68	0.36	6.06	0.06	1	1	10	0.00
Liguria	5	0.64	0.31	8.06	0.04	2	1	11	0.10
Moncao	6	0.33	0.33	7.14	0.00	1	1	10	0.00
Tuscany	5	0.31	0.31	8.06	0.00	1	1	11	0.00
Alcacier	5	0.31	0.31	8.06	0.00	1	1	11	0.00

Diversity and haplotype connectivity scores for the sampling locations pictured in Figure 5, ranked according to normalized average square-distance diversity score (AD*). The columns labeled with *AD*_min_, *AD*_max_, *HC*_min _and *HC*_max _contain the minimum/maximum score over all subsets containing the same number of haplotypes as found in the region.

As can be seen in Table 2, the two locations with highest AD* diversity scores are Landes and Pantelleria. In view of the HC* scores for these locations, this supports the melting-pot scenario, especially for the Landes location. Note that the bimodality of the GDS for the Landes location is also indicative of two clusters of haplotypes having low internal distances and high between cluster distances, which could also be regarded as a signature supporting a melting-pot scenario. However, the shape of the GDS for the Pantelleria location is somewhat less distinctive and so, in this case at least, the haplotype connectivity approach provides some useful additional information.

## Conclusions

We have presented a quantitative method to help shed light on the phylogeographic history of a species, in particular, for distinguishing between hot-spots and melting-pots of haplotypic diversity. The application of our method to the two data sets illustrates that our method should provide a useful addition to previously presented tools based on nested clade analysis and the GDS.

The algorithm for computing the haplotype connectivity of a collection of haplotypes can handle collections of several hundred haplotypes without difficulty. The computation of minimum and maximum haplotype connectivity scores over all subsets of a certain size, though still possible in polynomial time, is more demanding, especially computing the maximum as this involves the computation of minimum vertex separators in a graph. Although the (at least implicit) computation of such separators can probably not be avoided, for data sets where the haplotype connectivity must be computed for many subsets of different size, it could be interesting to develop a more efficient algorithm that preprocesses the distance matrix for the haplotypes so that *HC*_max_(*k*) can be quickly reported for any given *k*.

Our method depends on the haplotype distance and on the measure of diversity used for regions. However, based on experiments that we performed on the two data sets above (data not shown), we suspect that the impact of these two choices on the results will usually be quite small, at least for standard measures of distance and diversity. Also, since very low diversity scores will tend to yield low haplotype connectivity scores, we mainly recommend the use of our method only for regions yielding higher levels of haplotypic diversity (which is the case for both hot-spots and melting-pots). For example, for the Pine data above, consider the three Portuguese sampling locations Alcacier, Moncao and Leiria. In [39] it was suggested that there exists a glacial refugia of *Pinus pinaster *in Portugal. At least for Leiria our method supports this to some extent: In Table 2 we see that the normalized haplotype connectivity score is as small as possible while the normalized diversity score ranks third from top. But since, at the same time, the normalized diversity score is close to 0, it is somewhat less clear cut that this is indeed a hot-spot. Another potential difficulty arises from sampling issues. First note that the selection of a particular set of markers in a study can introduce a bias, and, second, the number of sampled haplotypes is often not the same for all regions. While the focus of this paper is on efficient algorithms for computing haplotype connectivity, to help interpret the significance of the scores obtained in a study, it would be interesting to investigate statistical properties of this quantity in future work. The computation of *HC*_min_(*k*) and *HC*_max_(*k*) can be viewed as first step towards a better understanding of the distribution of *HC*(*Y*) over all subsets *Y *⊆ *X *of size *k *for a given distance matrix *D*. Moreover, to place more emphasis on the geographical aspects of the problem, one could also consider the distribution of *HC*(*Y*) over only those subsets *Y *which satisfy some additional constraint such as, for example, insisting that any two haplotypes in *Y *are found within a certain maximum geographic distance related to the region sizes used in the study. In this paper, to address the sample-size bias, we have normalized our various scores with respect to the minimum and maximum scores that can be theoretically attained for a fixed number of haplotypes. If the measure of diversity used is such that computing the minimum and maximum is computationally too expensive, then averaging with respect to the number of haplotypes found in a region could be another possibility. However, some care would have to be taken since, as pointed out in [40], this might result in a normalized diversity score that could *increase *with the removal of a haplotype from a subset.

Another direction of potential interest is to extend our method to simultaneously take into account inter- and intra-species diversity. Many conservation approaches work by selecting species for conservation (see e.g. [41]). These species may be selected explicitly by allocating limited resources to them or implicitly by protecting the habitat containing them. In either approach species or regions are usually selected so as to protect maximal biodiversity. One example of such an approach that has recently attracted a lot of attention is the use of phylogenetic diversity [28,42].

The difficulty with such approaches is that they do not commonly take into account genetic diversity. For example, consider a situation where we might choose to conserve a species that makes a high contribution to phylogenetic diversity (since, for example, it is very different from any species that is likely to survive), but that has low genetic diversity. This low genetic diversity will limit the evolutionary potential of this species and its survival probability. It may therefore be better to conserve a different species that makes a lower contribution to phylogenetic diversity (since, for example, it is more closely related to another species with high survival probability) but has higher genetic diversity. It could be interesting to develop a framework that allows a combination of phylogenetic diversity and genetic diversity in reserve selection. One approach that might be worth exploring is using genetic diversity to allocate survival probabilities to species that could then be incorporated into Noah's Arc Problem frameworks for phylogenetic diversity [43]. This would allow the utilization of some of the algorithmic results that have been recently developed for solving this problem (cf. the survey in [42]).

With the large data sets that new high-throughput sequencing technologies are starting to deliver, our method will hopefully provide a fast and flexible way to analyze landscape scale genetic variation within species. In particular, it provides an efficient way to identify regions of probable long-term species persistence, a useful tool to identify regions of biodiversity conservation importance.

## Competing interests

The authors declare that they have no competing interests.

## Authors' contributions

BN implemented the algorithms for computing haplotype connectivity scores and carried out the analysis of the data sets. All authors participated in the design of the study, contributed to the writing of the manuscript, and read and approved the final version of the manuscript.
